# Computational Study to Identify the Effects of the KCNJ2 E299V Mutation in Cardiac Pumping Capacity

**DOI:** 10.1155/2020/7194275

**Published:** 2020-03-31

**Authors:** Da Un Jeong, Jiyeong Lee, Ki Moo Lim

**Affiliations:** ^1^Department of IT Convergence Engineering, Kumoh National Institute of Technology, Gumi 39177, Republic of Korea; ^2^Department of Medical IT Convergence Engineering, Kumoh National Institute of Technology, Gumi 39177, Republic of Korea

## Abstract

The KCNJ2 gene mutations induce short QT syndrome (SQT3) by directly increasing the *I*_K1_ current. There have been many studies on the electrophysiological effects of mutations such as the KCNJ2 D172N that cause the SQT3. However, the KCNJ2 E299V mutation is distinguished from other representative gene mutations that can induce the short QT syndrome (SQT3) in that it increased *I*_K1_ current by impairing the inward rectification of K^+^ channels. The studies of the electromechanical effects on myocardial cells and mechanisms of E299V mutations are limited. Therefore, we investigated the electrophysiological changes and the concomitant mechanical responses according to the expression levels of the KCNJ2 E299V mutation during sinus rhythm and ventricular fibrillation. We performed excitation-contraction coupling simulations using a human ventricular model with both electrophysiological and mechanical properties. In order to observe the electromechanical changes due to the expression of KCNJ2 E299V mutation, the simulations were performed under normal condition (WT), heterogeneous mutation condition (WT/E299V), and pure mutation condition (E299V). First, a single-cell simulation was performed in three types of ventricular cells (endocardial cell, midmyocardial cell, and epicardial cell) to confirm the electrophysiological changes and arrhythmogenesis caused by the KCNJ2 E299V mutation. In three-dimensional sinus rhythm simulations, we compared electrical changes and the corresponding changes in mechanical performance caused by the expression level of E299V mutation. Then, we observed the electromechanical properties of the E299V mutation during ventricular fibrillation using the three-dimensional reentry simulation. The KCNJ2 E299V mutation accelerated the opening of the *I*_K1_ channel and increased *I*_K1_ current, resulting in a decrease in action potential duration. Accordingly, the QT interval was reduced by 48% and 60% compared to the WT condition, for the WT/E299V and E299V conditions, respectively. During sustained reentry, the wavelength was reduced due to the KCNJ2 E299V mutation. Furthermore, there was almost no ventricular contraction in both WT/E299V and E299V conditions. We concluded that in both sinus rhythm and fibrillation, the KCNJ2 E299V mutation results in very low contractility regardless of the expression level of mutation and increases the risk of cardiac arrest and cardiac death.

## 1. Introduction

The inward rectifier potassium current (*I*_K1_) plays an important role in cardiac electrophysiological activity. If *I*_K1_ is abnormal, it can cause serious heart diseases such as chronic atrial fibrillation [1]. The KCNJ2 E299V mutation involved in the production of the KCNJ2 gene (Kir2.1) is a gain-of-function mutation that increases the magnitude of *I*_K1_ by impairing the rectification of the potassium channels [2]. Increased *I*_K1_ induces rapid repolarization of the action potential, thereby causing short QT syndrome (SQT3), which is a syndrome that the QT interval in the QRS complex of the electrocardiogram (ECG) is reduced [3]. SQT3 is a genetic disorder caused by KCNJ2 gene mutation that can cause structural and functional defects in the heart, leading to arrhythmia and sudden cardiac death [4, 5].

There have been many studies on the electrophysiological effects of the KCNJ2 E299V mutation on the heart. Domini et al. identified the possibility of atrial arrhythmia caused by the KCNJ2 D172N and KCNJ2 E299V mutations in SQT3 through the simulation study using virtual atrial models. They found that in two- and three-dimensional tissue models, both mutations make conduction wavelengths shorter by reducing the electrical refractoriness and conduction velocity, thereby stabilizing the reentrant wave [3]. Deo et al. confirmed the occurrence of ventricular fibrillation due to the E299V mutation that induces SQT3 [2]. They noted that the effect of the KCNJ2 E299V mutation is different from that of the KCNJ2 D172N mutation; the latter directly increases the amplitude of *I*_K1_, while the former indirectly increases the *I*_K1_ by impairing the rectification of the potassium channel. So far, there were no cases of ventricular tachycardia or ventricular fibrillation observed in patients with KCNJ2 E299V mutations, but it was reported that the E299V mutation may induce ventricular arrhythmia. Furthermore, similar to the V93I mutation, the electrophysiological changes due to the class-I antiarrhythmic agents used to treat atrial fibrillation by the E299V mutation can occur the reentrant waves through electrical excitation from the bundle branches of the Purkinje fibers, thereby inducing the ventricular arrhythmia [6].

Increased *I*_K1_ current due to KCNJ2 mutations including the E299V mutation reduces the heart rate and causes premature contractions of the ventricles [7, 8]. Adeniran et al. compared the effects of the SQT1 and SQT3 on a single-cell and on three-dimensional ventricular levels using an electromechanical coupling model. They reported that the ventricular contractility could be reduced by electrophysiological changes due to the SQT1 and SQT3. Furthermore, they suggested that there may be fragmentation between the end of the mechanical contraction and the beginning of the repolarization of the ventricle due to SQT syndrome [9]. They used the D172N mutation to identify the electromechanical effects of SQT3. However, as previously reported by Deo et al., the electrophysiological mechanisms of the D172N mutation and E299V mutation are different, and hence, the resulting contractility may vary.

Previously, we used a three-dimensional electromechanical ventricular model to predict the changes in the electrical and mechanical performance of the heart as a result of various pathological conditions including mutations [10–12]. In this study, we investigated the ventricular arrhythmogenesis and the mechanical contractility in relation to the expression level of KCNJ2 E299V mutation by using the electromechanical coupling model.

## 2. Materials and Methods

### 2.1. Cardiac Electrophysiological and Mechanical Model

**Figure 1:**
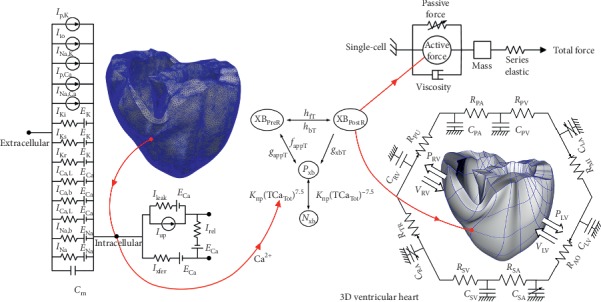
Schematic diagram of electromechanical coupling for single-cell and three-dimensional simulations. The left side of the diagram is an electrophysiology simulation. The electrical components symbolize the currents, pumps, and ion exchangers of the cell membrane to mimic the cardiac cells, as suggested by Ten Tusscher et al. “*I*” represents the ion currents, and “*E*” represents the equilibrium potentials of each ion. The right side of the diagram shows mechanical simulations at the single-cell and the 3D ventricular heart levels. The mechanical components come from myofilament models proposed by Rice et al. Mechanical model at the 3D ventricular heart level is coupled with the circulatory system; “*C*” and “*R*” represent compliance and resistance of each structure, respectively; PA, pulmonary artery; PV, pulmonary vein; LA, left atrium; MI, mitral valve; LV, left ventricles; AO, aortic valve; SA, systemic artery; SV, systemic vein; RA, right atrium; TR, tricuspid valve; RV, right ventricles; PU, pulmonary valve; “*P*” and “*V*” are pressure and volume, respectively (for more details, see text).

To study the changes in the electrophysiology and mechanical performance of the ventricles due to the KCNJ2 E299V mutation, we used the excitation-contraction coupling method. The diagrams of the electrophysiological and mechanical simulations are shown in Figure 1. The left-hand side of the diagram in Figure 1 represents the electrophysiological model, which is consisted of a lumped parameter circuit for simulating the myocardial cell. In the lumped parameter circuit, the electrical components symbolize the currents, pumps, and ion exchangers of the cell membrane and mimic the ion transport through the cell membrane and the sarcoplasmic reticulum within cardiac cells.

To simulate the ventricular cells, we modified and used the human ventricular ion model proposed by Ten Tusscher et al. [13]. To express the conduction of action potentials through myocardial cells as a numeric value, the following electrical conduction equation based on continuum mechanics was applied to the electrophysiological model:

(1) $$\begin{array}{c} \frac{\text{d}V}{\text{d}t}=-\frac{I_{\text{ion}}+I_{\text{stim}}}{C_{\text{m}}}. \end{array}$$

Here, *V* is the membrane potential of myocardial cells and *t* represents time. *I*_ion_ is the sum of all ion currents, *I*_stim_ is the current produced by an external stimulus, and *C*_m_ is the capacitance of the cell membrane. The sum of all ion currents is as follows:

(2) $$\begin{array}{c} I_{\text{ion}}=I_{\text{Na}}+I_{\text{K}1}+I_{\text{to}}+I_{\text{Kr}}+I_{\text{Ks}}+I_{\text{CaL}}+I_{\text{NaCa}}+I_{\text{NaK }}+I_{\text{pCa}}+I_{\text{pK}}+I_{\text{bCa}}+I_{\text{bNa}}. \end{array}$$

In this equation, *I*_Na_ is the current through Na^+^ channels. *I*_K1_, *I*_to_, *I*_Kr_, and *I*_Ks_ are currents through the K^+^ channels and represent the inward rectifier K^+^ current, transient outward K^+^ current, rapid delayed rectifier K^+^ current, and slow delayed rectifier K^+^ current, respectively. *I*_Ca,L_ is the L-type Ca^+^ current, *I*_NaCa_ is the current produced by the Na^+^-Ca^2+^ exchanger, and *I*_NaK_ is the current produced by the Na^+^-K^+^ exchanger. *I*_pCa_ and *I*_pK_ are the currents generated by the Ca^2+^ and K^+^ pumps, respectively. *I*_bCa_ and *I*_bNa_ are the background Ca^2+^ and Na^+^ currents, respectively. In the electrical model, “*E*” refers to the equilibrium potential; *E*_K_, *E*_Ca_, and *E*_Na_ are the equilibrium potentials of K^+^, Ca^2+^, and Na^+^ ions, respectively.

To express the electrical conduction of the action potentials in the three-dimensional ventricular tissues, we calculated the following equation consisted of the partial differential equation for electrical conduction through myocardial tissues and the ordinary differential equation for propagation of the electrical waveform:

(3) $$\begin{array}{c} \frac{\text{d}V}{\text{d}t}=-\frac{I_{\text{ion}}+I_{\text{stim}}}{C_{\text{m}}}+\frac{1}{\rho_{x}S_{x}C_{\text{m}}}\frac{\partial^{2}V}{\partial^{2}x^{2}}+\frac{1}{\rho_{y}S_{y}C_{\text{m}}}\frac{\partial^{2}V}{\partial^{2}y^{2}}+\frac{1}{\rho_{z}S_{z}C_{\text{m}}}\frac{\partial^{2}V}{\partial^{2}z^{2}}. \end{array}$$

Here, *ρ*_*x*_, *ρ*_*y*_, and *ρ*_*z*_ represent the cellular resistances along the *x*, *y*, and *z* axes, respectively, and *S*_*x*_*, S*_*y*_, and *S*_*z*_ represent the ratio of the volume to the surface along the *x*, y, and *z* axes, respectively.

We performed the excitation-contraction coupling simulation to observe the electrical and mechanical changes due to the KCNJ2 E299V mutation. For this purpose, we obtained the transient calcium information from the electrophysiological simulation and used it as the inputs to express the myocardial contraction derived by the calcium. We used the equation proposed by Ten Tusscher and Panfilov [14] to simulate the myocardial calcium mechanics, which is that calcium induces the contraction of the thin filaments via the calcium-induced calcium release (CICR) mechanism and thus produces myocardial tension:

(4) $$\begin{array}{c} \frac{{\text{dCa}}_{\text{itotal}}}{\text{d}t}=-\frac{I_{\text{Ca,L}}+I_{\text{b,Ca}}+I_{\text{p,Ca}}-2I_{\text{Na,Ca}}}{2V_{\text{C}}F}+I_{\text{leak}}-I_{\text{up}}+I_{\text{rel}},\\ \frac{{\text{dCa}}_{\text{srtotal}}}{\text{d}t}=\frac{V_{\text{c}}}{V_{\text{SR}}}\left(-I_{\text{leak}}+I_{\text{up}}-I_{\text{rel}}\right). \end{array}$$

In these equations, Ca_itotal_ and Ca_srtotal_ represent the total amounts of calcium in the cytoplasm and sarcoplasmic reticulum, respectively. *I*_leak_ is the leakage Ca^2+^ current of the junctional sarcoplasmic reticulum, *I*_up_ is the absorbed Ca^2+^ current in the network sarcoplasmic reticulum, and *I*_rel_ is the current induced by the release Ca^2+^ from the junctional sarcoplasmic reticulum.

The right-hand side of the diagram in Figure 1 represents the mechanical simulations at the single-cell and three-dimensional tissue levels. To simulate the cardiac mechanical contraction through the cross-bridge dynamics, we used the myocardial filament model proposed by Rice et al. [15], as shown in the middle of Figure 1:

(5) $$\begin{array}{c} F_{\text{active}}\left(x\right)={\text{SOVF}}_{\text{thick}}\left(x\right)\times \frac{x{\text{XB}}_{\text{PreR}}\times {\text{XB}}_{\text{PreR}}\times x{\text{XB}}_{\text{PostR}}\times {\text{XB}}_{\text{PostR}}}{x_{0}\times {\text{XB}}_{\text{PostR}}^{\text{Max}}}. \end{array}$$

SOVF_thick_ represents the single-overlap function of the thick filament. XB_PreR_ represents the prerotated state of the myosin head in relation to binding, which contributes to stiffness but does not generate force in the absence of net motion. XB_PostR_ is a strongly bound myosin head, which represents the isomerization that induces strain in the extensible neck region. *x*XB_PreR_ and *x*XB_PostR_ represent the average strain rate of XB_PreR_, and XB_PostR_, respectively. *x*_0_ is the average length of the cross-bridge. XB_PostR_^Max^ is the scaling factor of the postrotated state under optimal conditions such as high calcium activation, isosarcometric filaments, physiological temperature, and maximal single overlap of both thick filament and thin filament. *N*_xb_ and *P*_Xb_ are nonpermissive and permissive confirmations of regulatory proteins, respectively. *g*_xbT_ denotes the ATP-consuming detachment transition rate. *h*_fT_ and *h*_bT_ represent the forward and backward transition rates, respectively. *f*_appt_ is the cross-bridge attachment rate of transition to the first strongly bound state, and *g*_appT_ is the reverse rate. *K*_np_ and *K*_pn_ denote transition rates. Therefore, *K*_np_ (TCa_Tot_)^7.5^ is the forward rate of the nonpermissive-to-permissive transition, and *K*_pn_ (TCa_Tot_)^−7.5^ is the backward rate of permissive-to-nonpermissive transition.

### 2.2. Expression of KCNQ1 E229V Mutation

To simulate the change in *I*_K1_ current due to the KCNJ2 E299V mutation, the modified *I*_K1_ equation proposed by Deo et al. [2] was applied to the ventricular ion model. In this study, we formulated the following three *I*_K1_ current equations for the different expression levels of the KCNJ2 E299V mutation. These equations were used to simulate the *I*_K1_ currents in the wild-type condition without the mutation (WT), the heterogeneously expressed E299V mutation condition (WT/E299V), and the purely expressed E299V mutation condition (E299V), respectively:

(6) $$\begin{array}{c} \text{WT}\left(\text{control}\right)\text{:}I_{\text{K}1}=\frac{0.24731\left(V-E_{k}\right)}{0.86426+e^{0.0904\left(V-E_{k}\right)}}-0.06519,\\ \text{WT/E}229\text{V}:I_{\text{K}1}=\frac{0.11905\left(V-E_{k}+2.4\right)}{0.04092+e^{0.01732\left(V-E_{k}\right)}}-0.36212,\\ \text{E}299\text{V}:I_{\text{K}1}=0.06634\left(V-E_{k}+6.5\right)-2.44009\times 10^{-4}{\left(V-E_{k}\right)}^{2}-0.51383. \end{array}$$

We observed the electrophysiological changes due to the KCNJ2 E299V mutation at the single-cell and three-dimensional tissue levels and compared the resulting ventricular contractility according to the expression levels of mutation.

### 2.3. Three-Dimensional Human Ventricular Model

The three-dimensional human ventricular model used for the electrophysiological simulation is a finite element model consisting of a tetrahedral structure composed of 214,319 nodes and 1,061,379 elements. The three-dimensional ventricular model used for the mechanical contraction simulation is a finite element model consisting of a hexahedral structure composed of Hermite-based 14,720 nodes and 230 elements to represent the natural contraction of the ventricular tissue.

The elements of the ventricular model represent the different types of ventricular cells and are categorized as either endocardial cells, midmyocardial cells, or epicardial cells to imitate the heterogeneity of the ventricular tissue. The endocardial tissue conducts action potentials up to 2 mm from the endocardial surface to the internal tissue, while the epicardial tissue also conducts action potentials up to 2 mm but from the epicardial surface to the internal tissue. The midmyocardial tissue refers to the remaining inner area.

### 2.4. Simulation Protocols

In the single-cell simulation, we observed the electrophysiological changes in all three types of ventricular tissue cells in accordance with the expression levels of the KCNJ2 E299V mutation. The initial electrical conductivity of each ventricular cell was different. The electrical conductivity (*G*_Ks_) was 0.392 × 1.3 mS/*μ*F for the endocardial and epicardial cells and 0.073 mS/*μ*F for the midmyocardial cell. We demonstrated the cellular restitution of ventricular tissues in relation to the expression of the KCNJ2 E299V mutation using action potential duration restitution (APDr) curves. We compared the APDr-basic cycle length (BCL) curves and the APDr-diastolic interval curves of each ventricular cell under the WT, WT/E299V, and E299V conditions.

We simulated sinus rhythm using the three-dimensional human ventricular mesh with the Purkinje fiber. The Purkinje fiber was attached to the endocardial surface of the ventricular model and delivered the electrical stimulation to the endocardial tissue via the atrioventricular (AV) node. The sinus rhythm simulation was performed for 6 seconds with a BCL of 600 ms. Then, we compared the results for the last 600 ms, which is from 5,400 ms to 6,000 ms to observe the ventricles that reached a steady state. Furthermore, we determined the electrical activation time (EAT) and electrical deactivation time (EDT) of each cell to quantitatively observe the electrical phenomena associated with the expression levels of KCNJ2 E299V mutation. EAT is the time required for the first cell of the ventricle to activate, while EDT is the time required for the last cell of the ventricle to deactivate. In the mechanical simulation of the sinus rhythm, we calculated the left ventricular pressure-volume loop, the aortic pressure, the stroke volume, the ejection fraction, and the ATP consumption rate to quantitatively compare the relationship between electromechanical performance and the expression level of KCNJ2 E299V mutation.

In order to observe the electromechanical performance during the sustained reentry under WT, WT/E299V, and E299V conditions, we generated the reentrant wave using S1-S2 protocols at low conduction velocity. Then, we saved all the cellular state information obtained at the moment when the reentrant wave was stably maintained and used it as the initial condition in the sustained reentrant wave simulation at normal conduction velocity (70 cm/s). The sustained reentrant wave simulation was performed for 10 seconds. Similar to the sinus rhythm simulation, we extracted the transient calcium information from the reentry simulation and performed the excitation-contraction coupling simulation using the extracted calcium information. For the comparisons of WT, WT/E299V, and E299V conditions, we obtained the pressure of the systemic artery and the left ventricular pressure-volume loop.

## 3. Results and Discussion

### 3.1. Electrophysiological Response at the Cellular Level

**Figure 2:**
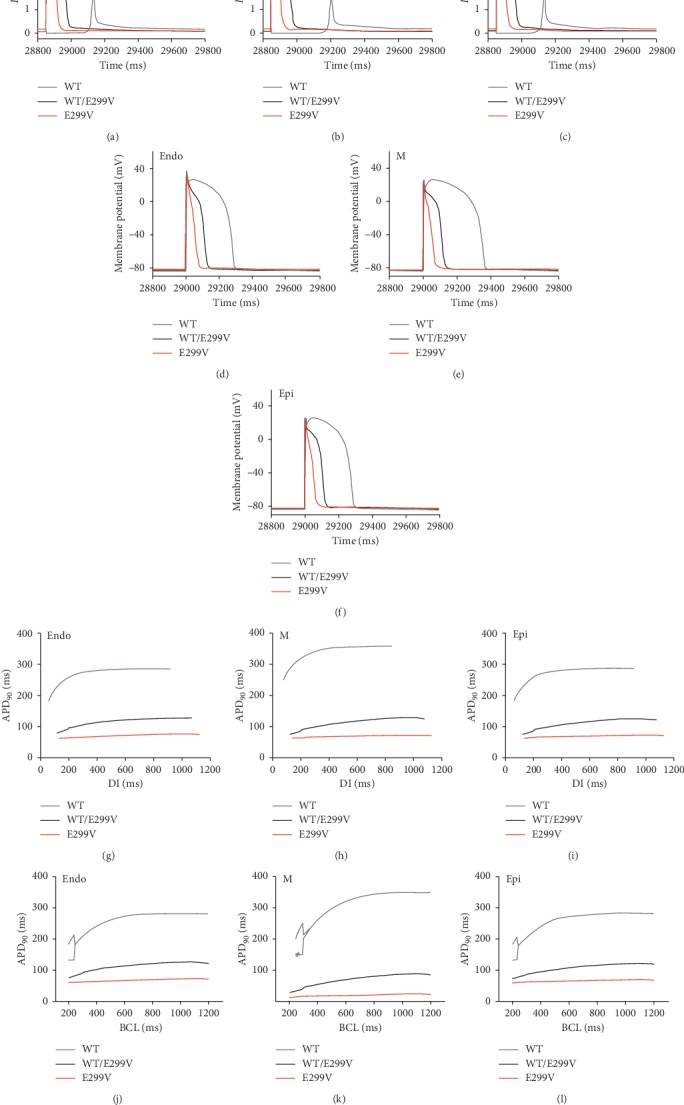
Electrophysiological results of human ventricular cells under the WT, WT/E299V, and E299V conditions. Current through rapid delayed rectifier K^+^ channel (a–c), membrane potential (d–f), action potential duration-diastolic interval (g–i), and action potential duration-basic cycle length (j–l) of the endocardial cell (Endo), the midmyocardial cell (M), and the epicardial cell (Epi).

At the cellular level, we observed changes in the *I*_K1_ current and the corresponding action potentials in the endocardial cell, midmyocardial cell, and epicardial cell due to the KCNJ2 E299V mutations (Figure 2). In all the ventricular cells, the KCNJ2 E299V mutation made *I*_K1_ channels open faster and remain active for longer periods compared to the WT condition (Figures 2(a)–2(c)). The activation time of the I_K1_ channel was the longest in the WT/E299V condition followed by the condition in which the E299V mutation was purely expressed (E299V). Accordingly, the increase of the *I*_K1_ current was greater under the E299V and WT/E299V conditions than under the WT condition. The *I*_K1_ current was the highest over 4 pA/pF under the E299V conditions. In the heterogeneous state of the E299V mutation (WT/E299V), the *I*_K1_ current was about 2.3 pA/pF, which was slightly greater than in WT condition (1.9 pA/pF).

In all ventricular cells, the repolarization of the action potential rapidly occurred in the presence of the E299V mutation (WT/E299V and E299V, Figures 2(d)–2(f)). Repolarization of the action potential occurred more rapidly when the E299V mutation was purely expressed (E299V) than when the mutation was heterogeneous (WT/E299V). APD was shorter under the E299V mutation conditions than the WT conditions. APD was the shortest at pure E299V mutation conditions (70 ms for endocardial cell, 70 ms for midmyocardial cell, and 67 ms for epicardial cell). Under the WT/E299V condition, APD was 123 ms for the endocardial cell, 123 ms for the myocardial cell, and 119 ms for the epicardial cell. Under the WT condition, APD of the endocardial cell, midmyocardial cell, and the endocardial cell was 308 ms, 390 ms, and 310 ms, respectively. Under the WT condition, there was a difference in APD depending on the type of ventricular tissue cell. However, there was little difference in APD under WT/E299V and E299V conditions.

We made the APDr curve to observe the generation of rate-dependent alternans and APD variation by reentrant wavefront under the WT, WT/E299V, and E299V conditions (Figures 2(g)–2(l)). Under the WT condition, the slope of the APDr curve was higher than 1 according to the diastolic interval, and alternans occurred at BCL 270 ms, 350 ms, and 260 ms in the endocardial, midmyocardial, and epicardial cells, respectively. However, under the WT/E299V and E299V conditions, the slope of the APDr curve was less than 1 in all ventricular cells according to the diastolic interval. Under the two E299V mutation conditions, the alternans did not occur, and the endocardial, midmyocardial, and epicardial cells remained stable.

### 3.2. Electromechanical Response as a Result of KCNJ2 E299V during Sinus Rhythm

**Figure 3:**
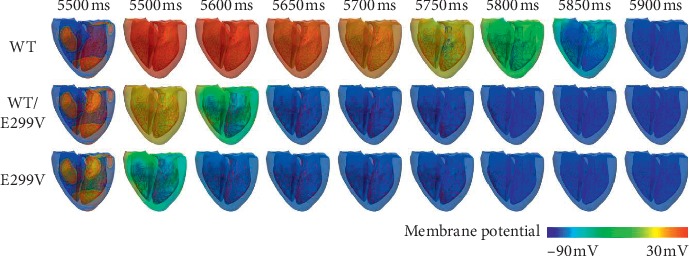
Contour of membrane potential during 600 ms sinus rhythm under each mutation condition. WT, wild type; WT/E299V, E299V mutation is moderately expressed heterogeneously; E299V, E299V mutation is purely expressed.

We observed the changes in electrophysiological conduction phenomena due to the KCNJ2 E299V mutation during the normal sinus rhythm of the 600 ms BCL through the Purkinje fibers from the AV node (Figure 3). The electrical depolarization time through the Purkinje fiber from the AV node was almost similar under all the three conditions, but there was a difference in the duration (APD) that electrical excitation of the ventricular tissue was maintained depending on the expression levels of E299V mutation. The duration of electrical excitation was the longest under the WT condition (repolarized at 5,850 ms) and the shortest under the pure E299V mutation condition (repolarized at 5,571 ms). In short, APD was 350 ms in WT condition, 121 ms in WT/E299V condition, and 61 ms in E299V condition. Under the three conditions, the conduction velocities were closer to each other at 67.4 ms for WT, 65.7 ms for WT/E299V, and 65.4 ms for E299V.

**Figure 4:**
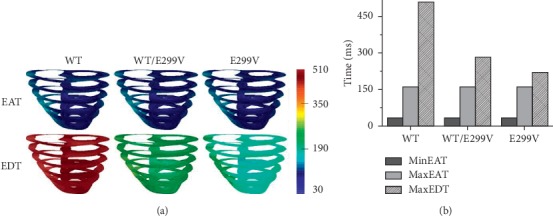
Electrical activation time (EAT) and electrical deactivation time (EDT) during 600 ms sinus rhythm. WT, wild type; WT/E299V, E299V mutation is moderately expressed heterogeneously; E299V, E299V mutation is purely expressed. (a) Horizontally sliced layers of EAT and EDT (from 30 ms to 510 ms). (b) Minimum and maximum values of EAT and maximum value of EDT.

We compared the EAT, the time at which the ventricular tissue activated, and the EDT, the time at which the ventricular tissue deactivated, during the 600 ms BCL in relation to the expression levels of the KCNJ2 E299V mutation (Figure 4). Since the electrical depolarization occurred at the same time through the Purkinje fiber, there was little difference in the EAT between the three conditions (WT, WT/E299V, and E299V). In contrast, the EDT was the fastest at 190 ms in the E299V condition with the shortest APD and the slowest at 510 ms in the WT condition with the longest APD. The EDT of the WT/E299V conditions was about 200 ms (Figure 4(a)).

The minimum EAT refers to the time period at which the ventricular tissue begins to get excited, and the maximum EAT represents the moment when the entire ventricular tissue is excited. Furthermore, the maximum EDT represents the moment when the repolarization of the entire ventricular tissue is over. Therefore, the difference between the maximum EAT and the minimum EAT means the QRS width in the ECG waveform, and the difference between the maximum EDT and the minimum EAT corresponds to the QT interval in the ECG waveform. There was no change in the QRS width as a result of the expression level of KCNJ2 E299V mutation (130 ms for all conditions). However, the QT interval was shorter under both the WT/E299V and E299V mutation conditions compared to the WT condition. The QT interval was 480 ms in the WT condition, 251 ms in the WT/E299V condition, and 191 ms in the E299V condition (Figure 4(b)).

**Figure 5:**
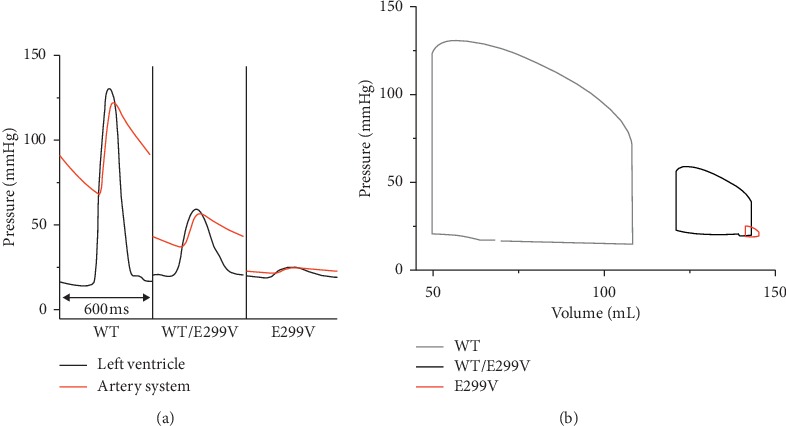
Mechanical response during 600 ms BCL of sinus rhythm under WT, WT/E299V, and E299V conditions. (a) Pressure of the left ventricle and artery system. (b) Pressure-volume loop of the left ventricle.

Through the excitation-contraction coupling simulation, we observed that the mechanical contractility changed during the sinus rhythm depending on the expression level of the KCNJ2 E299V mutation (Figure 5 and Table 1). When just one contraction had occurred in the ventricles, the ATP consumption rate under the WT condition was 47 s^−1^. Subsequently, it decreased to 18 s^−1^ under the WT/E299V condition and to 5 s^−1^ under the E299V condition.

Accordingly, the left ventricular and aortic pressures were significantly reduced in both WT/E299V and E299V conditions compared to the WT condition (Figure 5(a)). The maximum pressure of the left ventricle was 131 mmHg during the systole period under the WT condition. When the KCNJ2 E299V mutation was expressed, the left ventricular pressure of the systole period decreased and became to the pressure of the diastole period. The maximum pressure of the left ventricle was 59 mmHg in the WT/E299V condition and 25 mmHg in the E299V condition.

Furthermore, the end-systolic volume and the end-diastolic volume of the left ventricle were increased under the WT/E299V and the E299V conditions compared to those under the WT condition. Accordingly, the pressure-volume loops of the two E299V mutation conditions were shifted to the right (Figure 5(b)). The difference between the end-diastolic volume and the end-systolic volume corresponds to the stroke volume during the ventricular contraction cycle. In the sinus rhythm, the stroke volume was 59 mL under WT condition but decreased to 22 mL under WT/E299V condition and to 4 mL under the E299V condition. Consequently, the ejection fraction decreased depending on the expression level of KCNJ2 E299V mutation (54% for WT, 15% for WT/E299V, and 3% for E299V). The area of the pressure-volume loop refers to the stroke work, which is the amount of work the ventricle has done during the sinus rhythm. In the WT condition, the stroke work was 5,771 mmHg·mL but decreased in response to the expression level of KCNJ2 E299V mutation. In WT/E299V condition, the stroke work was significantly reduced to 712 mmHg·mL. The stroke work was the lowest at 16 mmHg·mL under the pure E299V mutation condition.

To quantitatively compare the mechanical performance during sinus rhythm, we computed the stroke work in relation to the ATP consumption. When the E299V mutation was moderately expressed (WT/E299V), the amount of work the left ventricle performed per unit ATP consumption was 67.7% less than that in the WT condition (124 s^−1^). It was reduced by 97.6% under the pure E299V mutation condition (3 s^−1^).

The cardiac output for 1 minute was 5,852 mL under the WT condition. However, as the contraction efficiency decreased in response to the expression levels of KCNJ2 E299V mutation, the cardiac output for 1 minute decreased to 2,209 mL under WT/E299V condition. Specifically, when the E299V mutation was purely expressed, the cardiac output for 1 minute was remarkably decreased to 378 mL.

### 3.3. Electromechanical Response as a Result of KCNJ2 E299V Mutation during Reentry

**Figure 6:**
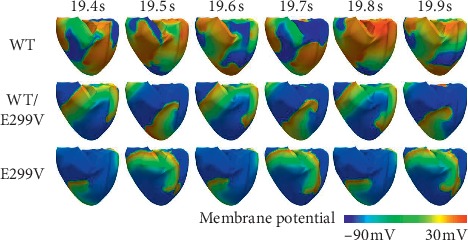
Membrane potential distribution of electrical reentrant wave during 0.6 s under WT, WT/E299V, and E299V mutation conditions.

We observed the electrical conduction in response to the expression levels of KCNJ2 E299V mutation when the reentrant wave was maintained via sustained reentry simulation (Figure 6). During the sustained reentry, the conduction wavelength of the reentrant wave decreased in both the WT/E299V and the E299V conditions as compared to the WT condition.

**Figure 7:**
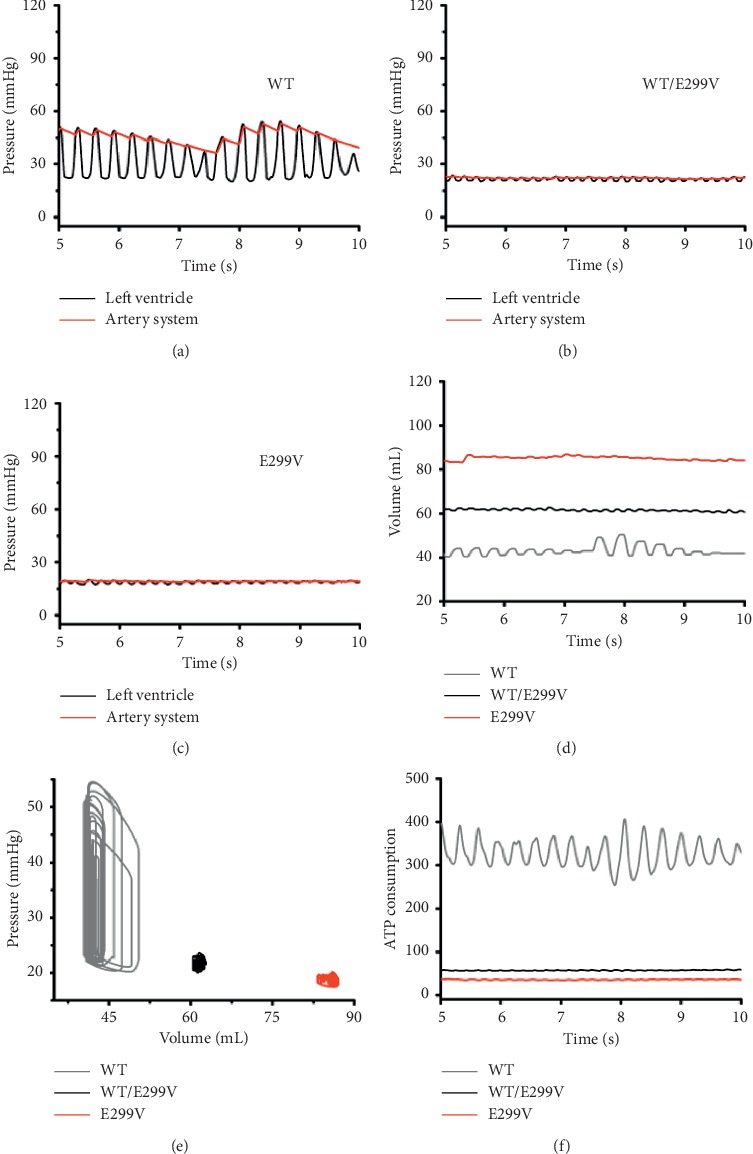
Cardiac response during sustained reentry for 5 s; pressure of left ventricle and artery system under WT (a), WT/E299V (b), and E299V (c) conditions; (d) volume of left ventricle; (e) pressure-volume loop of left ventricle; (f) ATP consumption rare.

Figure 7 shows the contractility due to the electrophysiological changes as a result of the expression levels of KCNJ2 E299V mutation during the ventricular tachyarrhythmia. Under all conditions of the expression level of the mutation, the pressures of the left ventricle and the aorta were lower than those in the sinus rhythm (47.32 mmHg for WT, 22.71 mmHg for WT/E299V, and 19.52 mmHg for E299V). The pressure fluctuations in the left ventricle and the aorta were lesser in WT/E299V condition than in the WT condition. In the E299V condition in which the E299V mutation was purely expressed, the pressures of the left ventricle and the aorta were almost not fluctuated (Figures 7(a)–7(c)).

Furthermore, the APD was reduced due to the KNCJ2 E299V mutation, which is similar to the results obtained from sinus rhythm simulation. Shortened APD decreased the amount of ATP consumed by the myocardium during the ventricular tachyarrhythmia (Figure 7(f)). Accordingly, in both the E299V mutation conditions, the contractility decreased, and the left ventricular volume increased. As the expression level of the KCNJ2 E299V mutation increased, the fluctuations of the left ventricular volume decreased (Figure 7(d)). That is, during the ventricular tachyarrhythmia, the E299V mutation caused little left ventricular contraction. The stroke volume was 5.42 mL in the WT condition, 0.68 mL in the WT/E299V condition, and 0.44 mL in the E299V condition.

## 4. Discussion

We observed the electrophysiological changes and the resulting mechanical performance according to the expression levels of the KCNJ2 E299V mutation during the sinus rhythm and the ventricular tachyarrhythmia. The main findings from this study are as follows:

1. In the electrophysiological simulation of a single cell, the KCNJ2 E299V mutation increased the density of *I*_K1_ and decreased APD in all ventricular tissue cells (endocardial cell, midmyocardial cell, and epicardial cell).
2. In the simulation of sinus rhythm, the ventricular repolarization time decreased, thereby shortening the maximum EDT by the expression levels of E299V mutation. The maximum EDT was the shortest when the E299V mutation was purely expressed.
3. The ejection fraction of the WT/E299V mutant ventricles was significantly lower than that of the WT condition. The pure E299V mutant ventricles failed to release blood during the sinus rhythm.
4. In the reentry simulation, the reentrant break-up occurred in the WT, WT/E299V, and E299V conditions. The conduction wavelength of the reentrant wave decreased as the degree of KCNJ2 E299V mutation increased. The left ventricles of both E299V mutant conditions (WT/E299V and E299V condition) showed little fluctuation in the pressure and volume and had a little mechanical contraction.

The KCNJ2 E299V gain-of-function mutation is expressed via the change in amino acid (from glutamic acid to valine) in the Kir2 channel. This mutation impairs the function of the inward rectifier channel resulting in the acceleration of the rectification and an increase in the *I*_K1_ current density. Increased *I*_K1_ induces the rapid repolarization of the action potential and reduces the depolarization period. The ventricular cell with shortened APD due to the KCNJ2 E299V mutation may not develop the alternans even at lower BCL conditions, whereas the ventricular cell with normal APD generates the alternans. These results correspond with the results of Wilder's advance research in 2004 [16].

Since the electrical stimulation is conducted simultaneously from the AV node and the Purkinje fibers, there may be no difference in the EAT representing the ventricular depolarization period under the WT, WT/E299V, and E299V conditions. However, the shortened depolarization period by the KCNJ2 E299V mutation reduces the maximum EDT, which is the repolarization time of the entire ventricles. Therefore, the QT interval obtained from the difference between the maximum EDT and the minimum EAT decreases in both E299V mutation conditions (WT/E299V and E299V). The short QT wave due to the KCNJ2 E299V mutation is the third form of the SQT3 mentioned in Priori et al.'s clinical study [4].

When the depolarization period is shortened by the KCNJ2 E299V mutation, the opening period of the L-type calcium channel is decreased, and the intracellular calcium concentration is also decreased. Reduced intracellular calcium concentration decreases the amount of calcium released by the CICR mechanism from the sarcoplasmic reticulum, thus resulting in a decrease in the calcium-binding to troponin for cross-bridge formation. The number of cross-bridges formed decreases and the amount of ATP consumed in the cross-bridge formation also decreases. Finally, the tension generated by the myofilament is reduced.

Reduced tension due to the KCNJ2 E299V mutation leads to a weakening of left ventricular contractility and an increase in the left ventricular volume. The increased internal area of the left ventricle causes a reduction in the pressures applied to the left ventricle and the aorta at the end of the systolic period according to Poiseuille's law. In the pressure-volume loops, the internal area is equal to the amount of work done by the left ventricles during contraction and is known as the stroke work. Accordingly, In the KCNJ2 E299V mutation conditions, the stroke volume and the stroke work of the left ventricle decrease and the ejection fraction goes down. These results are similar to those observed when the reentrant waves occur.

This study demonstrated the mechanical effects of the KCNJ2 E229V mutation as well as the electrophysiological effects of that during the ventricular sinus rhythm and fibrillation. Electrophysiological changes due to the KCNJ2 E299V mutation had a lethal mechanical effect on the contractile activity of the ventricles. The gain-of-function mutation including the KCNQ1 S140G [17], V241F [18], and G229D [19] is well known to be associated with the short QT wave in the ECG waveforms. We confirmed the SQT3 caused by the KCNJ2 E299V mutation can cause sudden cardiac death during ventricular fibrillation through the electromechanical simulation [20].

There are some limitations to our research. This study did not use any clinical trial data; instead, the results of the experiments were obtained using computer simulation. However, we used cell models [13, 14], organ models [21, 22], and methodologies [23, 24] that have been validated several times through the previous studies and the advanced researchers. Second, mechanical contraction may affect the electrophysiological phenomena, but in this study, only one-way coupling was used to observe mechanical behavior as a result of electrophysiological phenomena. However, these limitations do not affect the results of this study.

## 5. Conclusions

In this study, we reported the changes in the electrophysiological activity and corresponding mechanical behavior due to the KCNJ2 E299V mutation during sinus rhythm and ventricular tachyarrhythmia. The KCNJ2 E299V mutation can affect cardiac activity mechanically as well as electrophysiologically. Specifically, the KCNJ2 E299V mutation resulted in very low contractility during normal sinus rhythm regardless of the expression level of the mutation (heterogeneous, pure mutation). Furthermore, the mechanical performance of the left ventricle with KCNJ2 E299V mutation was very low under the sustained reentry condition. In conclusion, the KNCJ2 E299V mutation can increase the risk of cardiac death due to cardiac arrest during both sinus rhythm and tachyarrhythmia.

## Figures and Tables

**Table 1:** Cardiac mechanical responses during 600 ms BCL of sinus rhythm.

	Stroke volume (mL)	Ejection fraction (%)	Stroke work (mmHg·mL)	ATP consumption rate (s^−1^)	SW/ATP	Cardiac output (mL)
WT	59	54	5,771	47	124	5,852
WT/E299V	22	15	712	18	40	2,209
E299V	4	3	16	5	3	378

^*∗*^WT, wild type; WT/E299V, E299V mutation is moderately expressed heterogeneously; E299V, E299V mutation is purely expressed; SW, stroke work; ATP, adenosine triphosphate.

## Data Availability

The methods and results data used to support the findings of this study are included within the article.
